# Do Daily Fluctuations in Psychological and App-Related Variables Predict Engagement With an Alcohol Reduction App? A Series of N-Of-1 Studies

**DOI:** 10.2196/14098

**Published:** 2019-10-02

**Authors:** Olga Perski, Felix Naughton, Claire Garnett, Ann Blandford, Emma Beard, Robert West, Susan Michie

**Affiliations:** 1 Department of Behavioural Science and Health University College London London United Kingdom; 2 School of Health Sciences University of East Anglia Norwich United Kingdom; 3 UCL Interaction Centre University College London London United Kingdom; 4 Department of Clinical, Educational and Health Psychology University College London London United Kingdom

**Keywords:** apps, behavior change, excessive alcohol consumption, engagement, mHealth, n-of-1, time series analysis

## Abstract

**Background:**

Previous studies have identified psychological and smartphone app–related predictors of engagement with alcohol reduction apps at a group level. However, strategies to promote engagement need to be effective at the individual level. Evidence as to whether group-level predictors of engagement are also predictive for individuals is lacking.

**Objective:**

The aim of this study was to examine whether daily fluctuations in (1) the receipt of a reminder, (2) motivation to reduce alcohol, (3) perceived usefulness of the app, (4) alcohol consumption, and (5) perceived lack of time predicted within-person variability in the frequency and amount of engagement with an alcohol reduction app*.*

**Methods:**

We conducted a series of observational *N*-of-1 studies. The predictor variables were measured twice daily for 28 days via ecological momentary assessments. The outcome variables were measured through automated recordings of the participants’ app screen views. A total of nine London-based adults who drank alcohol excessively and were willing to set a reduction goal took part. Each participant’s dataset was analyzed separately using generalized additive mixed models to derive incidence rate ratios (IRRs) for the within-person associations of the predictor and outcome variables. Debriefing interviews, analyzed using thematic analysis, were used to contextualize the findings.

**Results:**

Predictors of the frequency and amount of engagement differed between individuals, and for the variables 'perceived usefulness of the app' and 'perceived lack of time', the direction of associations also differed between individuals. The most consistent predictors of within-person variability in the frequency of engagement were the receipt of a daily reminder (IRR=1.80-3.88; *P*<.05) and perceived usefulness of the app (IRR=0.82-1.42; *P*<.05). The most consistent predictors of within-person variability in the amount of engagement were motivation to reduce alcohol (IRR=1.67-3.45; *P*<.05) and perceived usefulness of the app (IRR=0.52-137.32; *P*<.05).

**Conclusions:**

The utility of the selected psychological and app-related variables in predicting the frequency and amount of engagement with an alcohol reduction app differed at the individual level. This highlights that key within-person associations may be masked in group-level designs and suggests that different strategies to promote engagement may be required for different individuals.

## Introduction

### Background

Excessive alcohol consumption is a public health priority and is implicated in substantial costs to the economy through lost productivity, crime, and health care costs [1,2]. Digital interventions, including websites, smartphone apps, and wearable devices, can increase access to behavioral support, have a low incremental cost once developed, and reduce stigma associated with help seeking in person [3-5]. Alcohol reduction apps have the added advantage of being available to users as and when needed. Some form of engagement, comprising both behavioral (eg, amount, depth, and frequency of app use) and experiential (eg, attention and interest) dimensions [6], is logically necessary for alcohol reduction apps to be effective [7,8]. Findings from an integrative literature review, in-depth interviews with potential users, theorizing within an interdisciplinary research team, and the development and evaluation of a novel self-report measure suggest that engagement with digital interventions can be defined as *“* a state-like construct which occurs each time a user interacts with a digital behavior change intervention with two behavioral (i.e. amount and depth of use) and three experiential (i.e. attention, interest and enjoyment) dimensions *”* (Perski et al, in press).

As observed levels of engagement with many digital interventions are considered too limited to support behavior change [9], efforts have been made to identify factors that predict engagement. Whether or not a user engages with a given digital intervention is likely to depend on its content (eg, behavior change techniques), how that content is delivered (eg, design features), the context in which the intervention is used (eg, who the users are and where they are using the intervention), whether or not the intervention succeeds in changing particular ‘mechanisms of action’ that mediate behavior change (eg, motivation and self-regulatory skills), and successful or unsuccessful behavior change (eg, the extent of alcohol reduction) [6]. To the authors’ knowledge, studies to date have typically focused on the identification of group-level predictors of engagement with digital interventions for alcohol reduction [6]. As strategies to increase engagement need to be effective for individuals [10,11], it is important to examine whether key predictors identified at the group level are also predictive at the individual level.

Published secondary analyses of data from randomized controlled trials (RCTs) of digital interventions for alcohol reduction have identified group-level predictors of engagement. These studies show that demographic (eg, being female, older, and more highly educated) [12-14], psychological (eg, higher levels of baseline motivation to change) [13,15], drinking (eg, lower baseline levels of alcohol consumption), [12,13,16] and app-related variables (eg, the receipt of proactive reminders) [17] predict the total frequency and amount of engagement.

Qualitative studies asking excessive drinkers to reflect on the factors they expect to be the most important for engagement with apps for alcohol reduction have identified the following: motivation to change, perceived personal relevance of the app (defined as the extent to which the user believes that the app is suited to their individual needs [18]), and perceived usefulness of the app (defined as the extent to which the individual believes that use of the app will help them achieve their goal(s) [19,20]). Although common themes were pulled out from these qualitative studies, agreement among potential users on what factors are expected to be most important for engagement was low [20]. Qualitative research has also been conducted with participants who disengaged before the completion of an RCT of a Web-based alcohol reduction intervention [13]. When retrospectively asked to reflect on why they disengaged from the intervention, users frequently mentioned perceived lack of time (eg, being too busy and having other priorities), dissatisfaction with the intervention (eg, poor usability and irrelevant content), and improvement in the condition (eg, feeling better).

As mentioned, quantitative studies examining predictors of engagement have typically relied on group-level designs, aggregating data across participants. However, individual-level interventions, including alcohol reduction apps, are designed to target within-person processes that lead to behavior change. Intervention strategies aimed at increasing engagement (eg, proactive reminders, rewards, and feedback) need to be effective for individuals. It is, therefore, important to examine whether associations identified at the group level are also identified at the individual level. The *N*-of-1 study design, also known as a single-case design, is ideally suited for the assessment of within-person processes. The *N*-of-1 design can be either observational or experimental and “...receives its name by virtue of its sample size: *N* is equal to one” [21].

Previous qualitative and quantitative research has relied on either prospective or retrospective (as opposed to real-time) self-reports of psychological processes; these are likely to be biased or inaccurate [22]. For example, when prospectively predicting what factors are expected to be most important for engagement, potential users tend to highlight app-related aspects, such as the presence of features that enhance motivation to change (eg, goal setting, self-monitoring, and proactive reminders) and perceived usefulness (eg, tailoring of content and rewards) [18,20]. However, when asked to retrospectively report on factors they thought contributed to their disengagement from a digital intervention, different aspects tended to be highlighted, such as perceived lack of time [13]. A data gathering method that overcomes the problems associated with both prospective and retrospective self-reports is ecological momentary assessment (EMA), which involves the repeated measurement of psychological processes in real time [23,24]. Methods for the statistical analysis of data from EMA and *N*-of-1 studies include correlational and time series analyses [25,26], with the latter being an underused approach to date.

### Objectives

This study used a series of *N*-of-1 studies, harnessing twice-daily EMAs for 28 days, and applied an innovative type of time series analysis to examine whether daily fluctuations in (1) the receipt of a reminder, (2) motivation to reduce alcohol, (3) perceived usefulness of the app, (4) alcohol consumption, and (5) perceived lack of time predicted within-person variability in the frequency (ie, number of log-ins) and amount (ie, time spent per log-in) of engagement with a theory- and evidence-informed alcohol reduction app, *Drink Less* [27,28]. This study aimed to provide a greater understanding of the temporal direction of the relationships under investigation by assessing predictor variables before the measurement of outcome variables.

## Methods

### Study Design

A prespecified study protocol and analysis plan can be found on the Open Science Framework [29]. A series of observational *N*-of-1 studies was conducted with twice-daily (ie, morning and evening) assessments of psychological and app-related predictor variables. The outcome variables were the objectively estimated frequency and amount of engagement with the *Drink Less* app, described in detail in the *Measures* section below. Although the subjective experience (eg, attention and interest) is also thought to be a key dimension of digital engagement ([6]; Perski et al, in press), only behavioral indicators of engagement (which can be measured automatically via participants’ app screen views) were considered in this study to minimize participant burden. Although it had been prespecified in the study protocol that the key outcome of interest was the frequency of engagement, a series of unplanned analyses with the variable 'amount of engagement' was also conducted. To help contextualize the quantitative findings, semistructured debriefing interviews were conducted over the phone after the 28-day study period.

### Participants and Sampling

The eligibility criteria are outlined in Textbox 1. Participants were excluded if they were not fluent English speakers. Recruitment was conducted on the Web via the research platform *Call for Participants*, social media (ie, Twitter), and an alcohol reduction charity’s mailing list. The recruitment materials stated that regular drinkers were invited to take part in a study on how people use alcohol reduction apps in their daily lives, which involved responding to twice-daily text messages for 28 days.

**Participant eligibility criteria.**

Eligibility criteria:

- Aged 18 years and older
- Owned an Apple iPhone capable of running iOS v.8.0 software or higher (ie, iPhone 4S or later models)
- Resided in or near London and willing to come to University College London for a briefing interview (to ensure adequate study commitment)
- Reported an Alcohol Use Disorders Identification Test score of ≥8, indicating excessive alcohol consumption [30]
- Was interested in using an app to reduce their drinking
- Was willing to set a goal to reduce their drinking
- Installed the *Drink Less* app and opened it at least once following the briefing interview
- Was willing to engage with the app daily for 28 days, recognizing that there may be occasional days where they would not engage with it [31]
- Was willing to respond to twice-daily text messages for 28 days
- Was willing to take part in a debriefing interview conducted over the phone

The number of observations (and not the number of participants) determines the statistical power in *N*-of-1 studies [32]. Each participant was asked to respond to twice-daily EMAs for 28 days, resulting in up to 56 data inputs per participant. The measurement frequency of 2 EMAs per day was informed by previous research conducted within the behavioral science domain [33]. The study duration of 28 days was selected as this is a common duration for digital alcohol reduction interventions [34]. As data were planned to be analyzed using generalized additive mixed models (GAMMs; see the *Data Analysis* section below), Monte Carlo simulations [35] estimated the statistical power achieved with a total of 56 data inputs. The power analysis, conducted in R, indicated that the study would have 80% power to detect an incidence rate ratio (IRR) of 1.8 for the association between ‘perceived usefulness of the app’ (predictor variable) and ‘frequency of engagement’ (outcome variable). Given the uncertainties regarding the distribution of model parameters, this power analysis should be interpreted with caution. See Table 1 for details about statistical assumptions used to inform the power analysis. To allow for a descriptive (but not inferential) comparison of potential between-person differences in the associations between the predictor variables and app engagement, a total of 8 participants was considered sufficient. As previous *N*-of-1 studies report up to 47% study dropout [33,36,37], we aimed to recruit an additional 50% of the target sample (ie, 12 participants).

**Table 1:** Statistical assumptions used to inform the simulation-based power analysis.

Considerations	Statistical assumptions and source of information (where available)
Model type	Generalized additive mixed model
Number of observations	Twice-daily ecological momentary assessments for a period of 28 days (ie, a total of 56 data inputs per participant)
Seasonality	No seasonality reflected by the day of the week the data were collected.
Distribution and point estimate (outcome variable)	The outcome variable (ie, frequency of engagement, operationalized as the number of app log-ins per measurement period) was assumed to follow a Poisson distribution with a mean of 11.7 log-ins per measurement period [28]. As the outcome variable represented count data, it was expected to follow a Poisson distribution. The mean of 11.7 log-ins was drawn from a group-level, factorial screening experiment of the *Drink Less* app [28], as this was judged to represent the best available data.
Distribution and point estimate (predictor variable)	The predictor variable (ie, perceived usefulness of the app), selected as a basis for the power analysis as data on the relationship of the other predictors and the frequency of engagement were lacking in the extant literature, was assumed to follow an autoregressive (AR) integrated moving average (MA) process with first-order autocorrelation, as it was expected that measurements would be similar to those taken 12 hours previously. We drew on the results from the between-person, factorial screening experiment of the *Drink Less* app, which assessed the variable ‘helpfulness of the app’ at 28-day follow-up. This variable was deemed to be conceptually similar to the target variable. It was, therefore, assumed that the mean level of the predictor variable would be 3.18 (SD 0.93) [28].

### Intervention

The *Drink Less* app is a stand-alone intervention designed to promote alcohol reduction in adults who drink excessively. The app is centered around a goal-setting module that allows users to select 1 or multiple weekly goals of their choice (eg, maximum number of units, alcohol-free days, spending on alcohol, or number of alcohol-attributed calories). The app includes 5 additional intervention modules: (1) normative feedback (ie, a visual gauge of how users’ drinking compares with that of others in the same gender and age group), (2) cognitive bias retraining (ie, a game that aims to help users retrain automatic approach/attentional biases toward alcohol-related cues), (3) self-monitoring and feedback (ie, an interactive calendar that allows users to record and visualize drinks consumed/alcohol-free days), (4) action planning (ie, a feature that explains the benefits of setting if-then rules and allows users to create, review, and edit these), and (5) identity change (ie, a feature that allows users to view pairs of positive and negative outcome expectancies, record video messages to watch at a later date, and identify and select values of importance to their identity). Details about how intervention content was selected [38,39], user feedback on an early version of the app [40], the development process [27], and the first evaluation of the app’s components in a randomized, factorial screening experiment [28] have been described in detail elsewhere. The *Drink Less* app allows users to set a daily reminder to open the app, which can be switched on or off and set to a suitable timing.

### Measures

The following data were collected at baseline to determine study eligibility and to describe the sample: (1) age, (2) gender, (3) type of work (ie, manual, nonmanual, or other), (4) whether the participants owned an iPhone capable of running iOS 8.0 software or higher (ie, iPhone 4S or later models), (5) whether the participants were residing in or near London and were willing to come to University College London (UCL) for a briefing interview, (6) alcohol consumption, measured using the Alcohol Use Disorders Identification Test [30], a 10-item measure of alcohol consumption, drinking behavior, and alcohol-related problems, which provides a score ranging from 0 to 40, with scores ≥8 indicating excessive alcohol consumption, (7) whether the participants were interested in using an app to reduce their drinking, (8) whether the participants were willing to set a goal to reduce their drinking, (9) whether the participants were willing to engage with the study app daily for 28 days, (10) whether the participants had previously used an alcohol reduction app and if so, which one, (11) whether the participants were willing to respond to the twice-daily text messages for 28 days, and (12) whether the participants were willing to take part in a poststudy interview conducted over the phone.

#### Ecological Momentary Assessments (Predictor Variables)

The following data were collected twice per day (ie, morning and evening):

- Motivation to reduce alcohol was measured by asking “How motivated are you currently to reduce your drinking?” The response options ranged from 1 to 7, with 1 indicating *not at all* and 7 indicating *extremely*.
- Perceived usefulness of the app was measured by asking “How useful do you currently think the *Drink Less* app is for you?” The response options ranged from 1 to 7, with 1 indicating *not at all* and 7 indicating *extremely*. The decision to focus on perceived usefulness of the app in this study was informed by a meta-analysis of 59 studies indicating that the variable ‘perceived usefulness’ is consistently associated with behavioral intentions to use technology (*r*=.59) [41]; less is known about the relationship between the variable ‘perceived relevance’ and key outcome variables. This variable captured participants’ beliefs about the app’s usefulness and was considered in the absence of any objective effectiveness data from a confirmatory RCT.
- Alcohol consumption was measured by asking “How many drinks containing alcohol have you had in the past 12 hours?” Participants were instructed to input integers only (ie, whole drinks).
- Perceived lack of time was measured by asking participants “To what extent do you currently have time for the *Drink Less* app?” The response options ranged from 1 to 7, with 1 indicating *I do not have any time for the app* and 7 indicating *I have lots of time for the app*.

An additional predictor variable, tailored to participants’ preferences, was as follows:

- Whether or not a proactive reminder was received during each 12-hour measurement period; this variable was coded 1 if a reminder was received and 0 if it was not received. A maximum of 1 reminder could be received every 24 hours, and the frequency and the timing of the reminders did not change during the course of the study.

#### Outcome Variables

App screen views were automatically recorded, stored in a Web-based database, and extracted using the free python library *pandas* to derive the outcome variables frequency of engagement and amount of engagement. The variable 'frequency of engagement' was operationalized as the number of log-ins during each 12-hour measurement period, with a log-in defined as a new screen view following at least 30 min of inactivity [42]. The variable 'amount of engagement' was derived by calculating the time spent (in seconds) per 12-hour measurement period. For descriptive purposes, the variable 'depth of engagement' was also derived, which was operationalized as the number of app components accessed per 12-hour measurement period, indexed as a proportion of the number of available app components. However, as depth of engagement was strongly correlated with amount of engagement for all participants, no inferential analyses were conducted using this variable.

### Procedure

Participants who expressed an interest in taking part were asked to read the participant information sheet, provide informed consent, and fill out the Web-based screening questionnaire hosted via Qualtrics [43]. Eligible participants were invited to a briefing interview at UCL where they were asked to reread the information sheet and were consented. Participants were asked to download the *Drink Less* app, briefly explore it, and set at least 1 weekly alcohol reduction goal of their choice. They were also asked if they wanted to switch the daily reminder on or off and if applicable, select a suitable timing for these. After having explored the app, participants were asked to complete a brief survey on their phone, which fetched their unique user identity document, generated by the *Drink Less* app. This information enabled the researchers to match participants to their app screen views and, hence, derive the outcome variables. Participants were asked a few questions about their expected app use and what they were hoping to achieve using the app (not reported). They were subsequently asked to familiarize themselves with the daily EMA questions and response options and practice inputting their responses to the 4 questions into a single text message. They were also asked to select a suitable timing for the EMAs. In the morning, participants were asked to select a time between 6 am and 10 am and in the evening, between 6 pm and 10 pm, ensuring that the selected time points did not fall earlier/later than their usual morning and evening bedtimes, respectively. No particular instructions about app engagement were provided other than that participants were expected to engage with the app at least once daily for 28 days, recognizing that there might be occasional days when they would not engage with it. Participants were told that they had to respond to at least 70% of the text messages and take part in the debriefing interview to receive any payment. They were also asked to notify the study team if they decided to change the timing of the daily reminder, so that this could be accounted for in the statistical analyses. The briefing interviews lasted between 29 and 63 min.

Participants were then asked to respond to the twice-daily text messages for 28 days, sent manually from an iPhone 6S by the first author. The first text message was sent the morning after the briefing interview. When a response was received, participants were sent the following standard response: “Thank you for your responses!” Participants also received weekly updates via text message about their survey response rate to encourage adherence to the study materials (eg, “Hi X! Thank you for completing the first week of the study. You have responded to X/14 text messages. Keep up the good work!”). If the text messages were not received in the expected format, participants received a standard reply with instructions for how to input the responses (ie, “Hi X! It appears that your responses are not in the expected format. Please enter your responses as follows: a=X; b=X; c=X; d=X”).

After 28 days, participants were invited to take part in a debriefing interview conducted over the phone, during which they were asked about their experiences of engaging with the *Drink Less* app. The interviews lasted between 25 and 47 min.

Participants were paid £0.50 per data input (ie, a maximum of £28), in addition to £32 upon study completion, resulting in a possible total of £60. This was paid to participants in the form of a shopping voucher.

### Data Analysis

Guided by published research in the behavioral science domain [33,36,37], in time series with >5% missing data, multiple imputation was conducted using an expectation-maximization with bootstrapping algorithm via the R package *Amelia II*. Data were imputed separately for each dataset (ie, each participant). A polynomial time trend (ie, linear or quadratic) was included if this was found to improve the precision of the imputed data points. This was decided upon by examining the 95% CIs of the means of the imputed data points. A total of 5 imputed datasets were created per dataset with missing values, which were combined before conducting further statistical analyses using Rubin rules [33,36,37].

Descriptive statistics were calculated for each participant. Time series analyses were conducted using the R package *mgcv*: GAMMs were fitted to estimate IRRs for the associations between the predictor and the outcome variables. The IRR is a measure of relative difference and can, in this particular context, be interpreted as the relative frequency or amount of engagement for the different levels of the predictor variables. The GAMM is a type of multilevel model that has previously been applied to data from *N*-of-1 studies [44]. GAMMs are particularly well suited to the modeling of time series data with 1 level of measurement (ie, repeated measurements nested within 1 individual), as they accommodate the inclusion of autocorrelation [44]. The analyses proceeded in a number of stages using a backward selection procedure:

- As the outcome variables represented counts, data were first assessed for overdispersion (ie, when the variance is greater than the mean). If there was evidence for overdispersion, a quasi-Poisson distribution (rather than a Poisson distribution) was specified.
- As repeated measures taken from the same individual are often correlated, data from *N*-of-1 studies typically violate the assumption of independence of observations. Autocorrelation was therefore assessed through the autocorrelation function and the partial autocorrelation function. Evidence of first-order autocorrelation in this study would mean that measurements were significantly correlated with those taken 12 hours previously.
- A full model including all predictor variables was first fitted to determine the most appropriate autocorrelation structure for each participant. Model fit was compared using Akaike’s Information Criterion [45]. Although the *a priori* power analysis did not take account of the adjustment for seasonality or moving average (MA) terms, it was determined *a posteriori* that adjusting for the day of the week through the inclusion of a cyclic cubic smoothing term significantly improved the model fit for all participants and that the inclusion of an MA term improved the model fit for some participants.
- For visualization purposes, univariable models for each predictor variable were fitted for each participant, carrying forward the most appropriate autocorrelation structure and MA terms from the previous step.
- Parsimonious multivariable models were subsequently built through the stepwise elimination of redundant terms. The predictor variables were sequentially varied to arrive at a best-fitting model for each participant.

#### Debriefing Interviews

Telephone interviews were audio-recorded, transcribed verbatim by the first author, and analyzed using inductive thematic analysis [46], which involved the following steps: (1) data familiarization, (2) initial code generation, (3) searching for themes, (4) reviewing the themes, (5) defining and naming the themes, and (6) producing the report. Data were coded by the first author and reviewed by the third author. New inductive codes were labeled as they were identified during the coding process. Codes were subsequently reviewed one by one and systematically organized into themes.

### Ethical Approval

Ethical approval was granted by UCL’s Computer Science Departmental Research Ethics Chair (Project ID: UCLIC/1617/004/Staff Blandford HFDH). Personal identifiers were removed and anonymized data were stored securely on a password-protected computer. Participants’ contact details were stored separately in a locked cabinet. The subscriber identification module card used to deliver the daily text messages was wiped upon completion of the data collection.

## Results

### Participants

Of the 22 participants who completed the Web-based screening questionnaire, 11 met the inclusion criteria and were invited to take part. Of these, 1 was unable to initiate the 28-day study during the planned study period. In total, 10 participants took part between June 29 and August 9, 2018. A participant broke their phone 14 days into the study and redownloaded the app onto a new phone without notifying the researchers. Owing to these technical issues, the new phone’s app screens failed to sync with the database, and hence, the outcome data for the last 14 days of the study were lost. This participant was therefore excluded from the inferential analyses, but descriptive statistics were calculated for all 10 participants. Participants’ characteristics are summarized in Table 2.

**Table 2:** Participants’ demographic, drinking, and app-related characteristics.

Participant (P) identifier	Gender	Age (years)	Occupational status	Alcohol Use Disorders Identification Test	Past use of an alcohol reduction app	Past use of the *Drink Less* app
P1	Female	28	Nonmanual	16	No	No
P2	Female	20	Other	10	No	No
P3	Female	25	Nonmanual	30	No	No
P4	Female	18	Other	12	No	No
P5	Male	21	Other	22	No	No
P6	Female	31	Nonmanual	8	No	No
P7	Female	23	Nonmanual	12	Yes	Yes
P8	Female	30	Nonmanual	11	No	No
P9	Female	28	Other	23	Yes	No
P10	Female	26	Nonmanual	10	No	No

### Descriptive Statistics

A total of 8 participants (8/10, 80%) opted to have the daily reminder switched on. Overall, participants displayed high compliance with the daily text messages (mean 93%; SD 5.8%), with the number of missing responses varying from 0% to 16% (see Table 3). Descriptive statistics for the predictor variables are displayed in Table 4.

Participants’ total number of log-ins ranged from 10 to 69 (see Table 5). The total depth of engagement over the 28-day study period ranged from 14% (ie, accessing 1 of the app’s 7 components) to 86% (ie, accessing 6 of the app’s 7 components), and the total amount of engagement ranged from 4 min and 24 seconds to 70 min and 14 seconds. See Multimedia Appendix 1 for plots of participants’ frequency and amount of engagement over the course of the study.

**Table 3:** Compliance with the twice-daily ecological momentary assessments.

Participant (P) identifier	Compliance (N=56), n (%)	Timing of text messages	Daily reminder switched on/off	Timing of daily reminder
P1	56 (100)	10 am/pm	On	10 am
P2	55 (98)	10 am/pm	On	1 pm
P3	50 (89)	7:30 am/pm	On	4 pm
P4	49 (88)	10 am/pm	On	11 am
P5	55 (98)	9:30 am/pm	Off	—^a^
P6	47 (84)	10 am/pm	On	10 am
P7	48 (86)	9 am/pm	On	9 am
P8	51 (91)	10 am/pm	Off	—
P9	56 (100)	10 am/pm	On	10:30 am
P10	54 (96)	10 am/pm	On	9 am

^a^Not applicable.

**Table 4:** Descriptive statistics for the predictor variables.

Participant (P) identifier	Motivation to reduce alcohol		Perceived usefulness of the app		Alcohol consumption (drinks)		Perceived lack of time	
	Mean^a^ (SD)	Range	Mean^a^ (SD)	Range	Mean^a^ (SD)	Range	Mean^a^ (SD)	Range
P1	5.3 (1.1)	3-7	5.4 (0.8)	4-7	2.1 (2.8)	0-10	6.1 (1.2)	3-7
P2	6.3 (1.1)^b^	3-7	6.3 (1.1)^b^	3-7	0.1 (0.5)^b^	0-3	4.6 (2.2)^b^	1-7
P3	5.2 (0.9)^b^	4-7	5.3 (1.1)^b^	3-7	1.2 (1.3)^b^	0-5	4.5 (1.0)^b^	2-7
P4	4.1 (1.6)^b^	1-7	2.4 (1.3)^b^	1-5	0.1 (0.8)^b^	0-4	4.9 (1.8)^b^	2-7
P5	3.6 (1.0)^b^	2-6	3.6 (1.2)^b^	1-7	1.2 (1.7)^b^	0-8	3.9 (0.9)^b^	2-7
P6	5.6 (0.7)^b^	4-7	4.4 (0.6)^b^	4-6	0.3 (0.8)^b^	0-3	4.4 (0.7)^b^	3-7
P7	4.1 (1.2)^b^	1-6	3.2 (0.9)^b^	2-5	1.1 (2.1)^b^	0-6	2.8 (1.6)^b^	1-6
P8	5.9 (0.5)^b^	4-7	6.1 (0.9)^b^	4-7	0.4 (0.9)^b^	0-4	2.2 (1.4)^b^	1-5
P9	4.3 (1.9)	1-7	1.9 (0.9)	1-5	3.9 (4.3)	0-14	6.0 (1.3)	2-7
P10	5.3 (1.6)^b^	1-7	4.8 (1.0)^b^	1-6	1.9 (2.9)^b^	0-9	5.5 (1.0)^b^	3-7

^a^Mean levels for the predictor variables over the 56 12-hour measurement periods.

^b^For participants with missing data, means and standard deviations for the complete datasets (after multiple imputation) were computed using Rubin rules.

**Table 5:** Descriptive statistics of participants’ frequency, amount, and depth of engagement with the *Drink Less* app.

Participant (P) identifier	Log-ins over the 28-day study			Total amount of engagement over the 28-day study (minutes:seconds)	Amount of engagement per measurement period (minutes:seconds)		Total depth of engagement over the 28-day study (%)	Depth of engagement per measurement period (%), mean (SD)
	Total number	Mean (SD)	Range		Mean (SD)	Range		
P1	39	0.7 (0.7)	0-3	23:11	00:26 (00:53)	00:00-04:12	71	10 (12)
P2	47	0.8 (0.8)	0-4	60:43	01:06 (02:33)	00:00-16:32	86	20 (20)
P3	35	0.6 (0.6)	0-2	13:12	00:14 (00:27)	00:00-02:19	57	10 (11)
P4	10	0.2 (0.5)	0-2	04:24	00:05 (00:18)	00:00-01:29	43	3 (8)
P5	42	0.8 (0.7)	0-3	18:20	00:20 (00:29)	00:00-01:11	29	11 (11)
P6	31	0.6 (0.6)	0-2	39:19	00:42 (85.42)	00:00-08:12	57	9 (11)
P7	64	1.1 (0.9)	0-3	19:14	00:21 (00:27)	00:00-02:44	14	10 (6)
P8	69	1.2 (0.9)	0-3	70:14	01:09 (02:01)	00:00-10:47	43	17 (13)
P9	34	0.6 (0.7)	0-2	35:26	00:38 (02:04)	00:00-13:40	43	9 (11)
P10	—^a^	—	—	—	—	—	—	—

^a^Due to a technical issue, data were lost for P10.

### Predicting the Frequency and the Amount of Engagement

The results from the univariable GAMMs can be found in Multimedia Appendix 2. For visualization purposes, plots of the IRRs and 95% CIs are depicted in the below figures. Table 6 reports the results from the multivariable GAMMs. In some cases, results from the univariable and multivariable models differed. Hence, interpretations are based on both uni- and multivariable analyses.

**Table 6:** Incidence rate ratios for the associations between the predictor and the outcome variables for each participant (P) in the multivariable generalized additive mixed models.

Participant		Frequency of engagement^a^		Amount of engagement^a^	
		Incidence rate ratio (IRR) (95% CI)	*P* value	IRR (95% CI)	*P* value
**P1**					
	Reminder	1.80_2,1_^b^ (1.19-2.74)	*.01* ^c^	—^d^	—
	Motivation to reduce alcohol	1.14_2,1_ (1.02-1.27)	*.02*	1.12_0,0_ (0.68-1.83)	.65
	Perceived usefulness of the app	0.82_2,1_ (0.68-0.99)	*.04*	—	—
	Alcohol consumption	—	—	—	—
	Perceived lack of time	0.93_2,1_ (0.86-1.02)	.15	—	—
**P2**					
	Reminder	1.99_1,0_ (0.67-5.94)	.22	—	—
	Motivation to reduce alcohol	—	—	—	—
	Perceived usefulness of the app	—	—	—	—
	Alcohol consumption	1.50_1,0_ (1.16-1.93)	*.003*	2.38_1,0_ (1.65-3.43)	*<.001*
	Perceived lack of time	1.13_1,0_ (1.01-1.25)	*.03*	—	—
**P3**					
	Reminder	—	—	4.31_0,0_ (1.73-10.73)	*.003*
	Motivation to reduce alcohol	0.89_1,0_ (0.67-1.19)	.45	—	—
	Perceived usefulness of the app	—	—	—	—
	Alcohol consumption	—	—	1.38_0,0_ (1.11-1.73)	*.006*
	Perceived lack of time	—	—	1.19_0,0_ (0.79-1.77)	.40
**P4^**e**^**					
	Reminder	—	—	—	—
	Motivation to reduce alcohol	1.88_0,0_ (1.22-2.91)	*.005*	2.03_0,0_ (1.72-2.40)	*<.001*
	Perceived usefulness of the app	—	—	137.33_0,0_ (49.45-381.34)	*<.001*
	Alcohol consumption	—	—	—	—
	Perceived lack of time	—	—	0.20_0,0_ (0.14-0.29)	*<.001*
**P5^**f**^**					
	Motivation to reduce alcohol	—	—	—	—
	Perceived usefulness of the app	1.42_2,2_ (1.15-1.75)	*.002*	1.39_0,0_ (1.06-1.82)	*.02*
	Alcohol consumption	—	—	—	—
	Perceived lack of time	1.08_2,2_ (0.81-1.43)	.60	—	—
**P6**					
	Reminder	3.88_2,0_ (1.37-11.03)	*.01*	—	—
	Motivation to reduce alcohol	1.07_2,0_ (0.93-1.21)	.35	3.45_0,0_ (1.34-8.83)	*.01*
	Perceived usefulness of the app	1.12_2,0_ (0.94-1.34)	.21	—	—
	Alcohol consumption	0.92_2,0_ (0.83-1.02)	.13	—	—
	Perceived lack of time	0.77_2,0_ (0.61-0.97)	*.03*	1.24_0,0_ (0.71-2.17)	.45
**P7**					
	Reminder	3.26_1,0_ (2.15-4.96)	*<.001*	—	—
	Motivation to reduce alcohol	—	—	1.67_0,0_ (1.16-2.40)	*.008*
	Perceived usefulness of the app	—	—	0.52_0,0_ (0.33-0.80)	*.005*
	Alcohol consumption	—	—	—	—
	Perceived lack of time	—	—	—	—
**P8^**f**^**					
	Motivation to reduce alcohol	—	—	—	—
	Perceived usefulness of the app	—	—	—	—
	Alcohol consumption	0.85_1,0_ (0.67-1.09)	.20	0.82_0,0_ (0.47-1.43)	.50
	Perceived lack of time	—	—	1.33_0,0_ (0.97-1.82)	.08
**P9**					
	Reminder	—	—	—	—
	Motivation to reduce alcohol	—	—	1.20_1,1_ (0.92-1.58)	0.18
	Perceived usefulness of the app	1.38_1,0_ (1.24-1.53)	*<.001*	1.67_1,1_ (1.22-2.29)	*.002*
	Alcohol consumption	—	—	—	—
	Perceived lack of time	—	—	4.77_1,1_ (1.09-20.79)	*.04*

^a^All models were adjusted for the day of the week using a cyclic cubic smoothing term.

^b^Numbers in subscript indicate the lags of autoregressive (AR) and moving average (MA) terms, respectively. A lag value of 0 indicates that an AR or an MA term was not included.

^c^*P* values significant at the .05 level are highlighted in italics.

^d^Indicates that a predictor variable was not included in the best-fitting model.

^e^For P4, generalized additive mixed models would not converge. Therefore, generalized additive models were fitted.

^f^As P5 and P8 opted out of receiving the daily reminder, this variable did not apply to these 2 participants.

#### Daily Reminder

**Figure 1:**
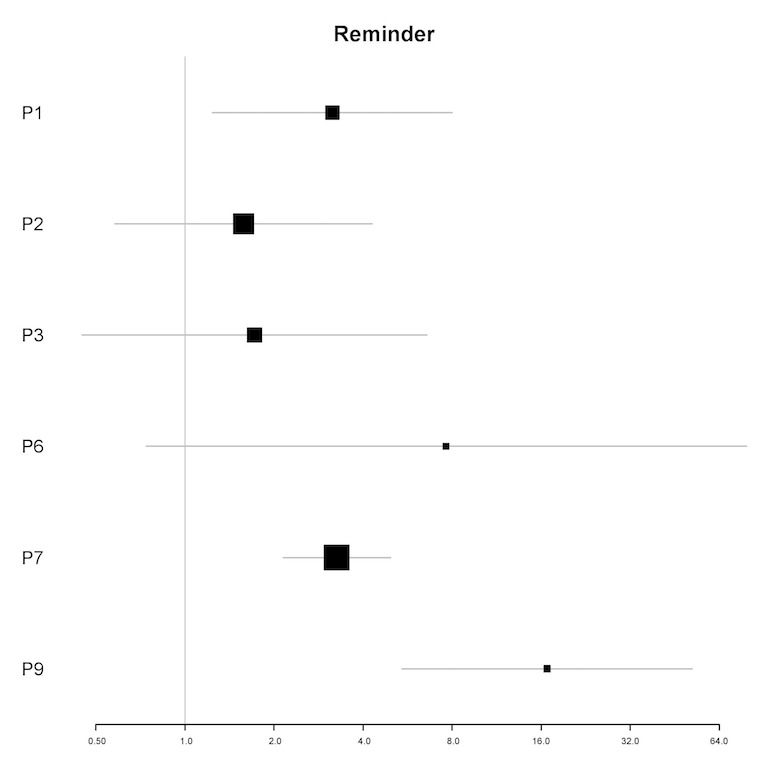
Plot of incidence rate ratios and 95% CIs (x-axis) for the association of the daily reminder and the frequency of engagement for each participant (y-axis) in univariable analyses. The vertical line indicates parity; 95% CIs that cross the line of parity indicate nonsignificant incidence rate ratios. For P4, the univariable model did not converge. P4 is hence not included in this plot. P: participant.

In univariable analyses, the daily reminder was a significant predictor of the frequency of engagement for 3 participants (P1, P7, and P9; see Figure 1). In multivariable analyses, the daily reminder was a significant predictor for 3 participants (IRR=1.80-3.88; all *P*<.05). For these participants (P1, P6, and P7), the receipt of a reminder was associated with an 80% to 288% increase in the number of log-ins in the next 12 hours (see Table 6).

**Figure 2:**
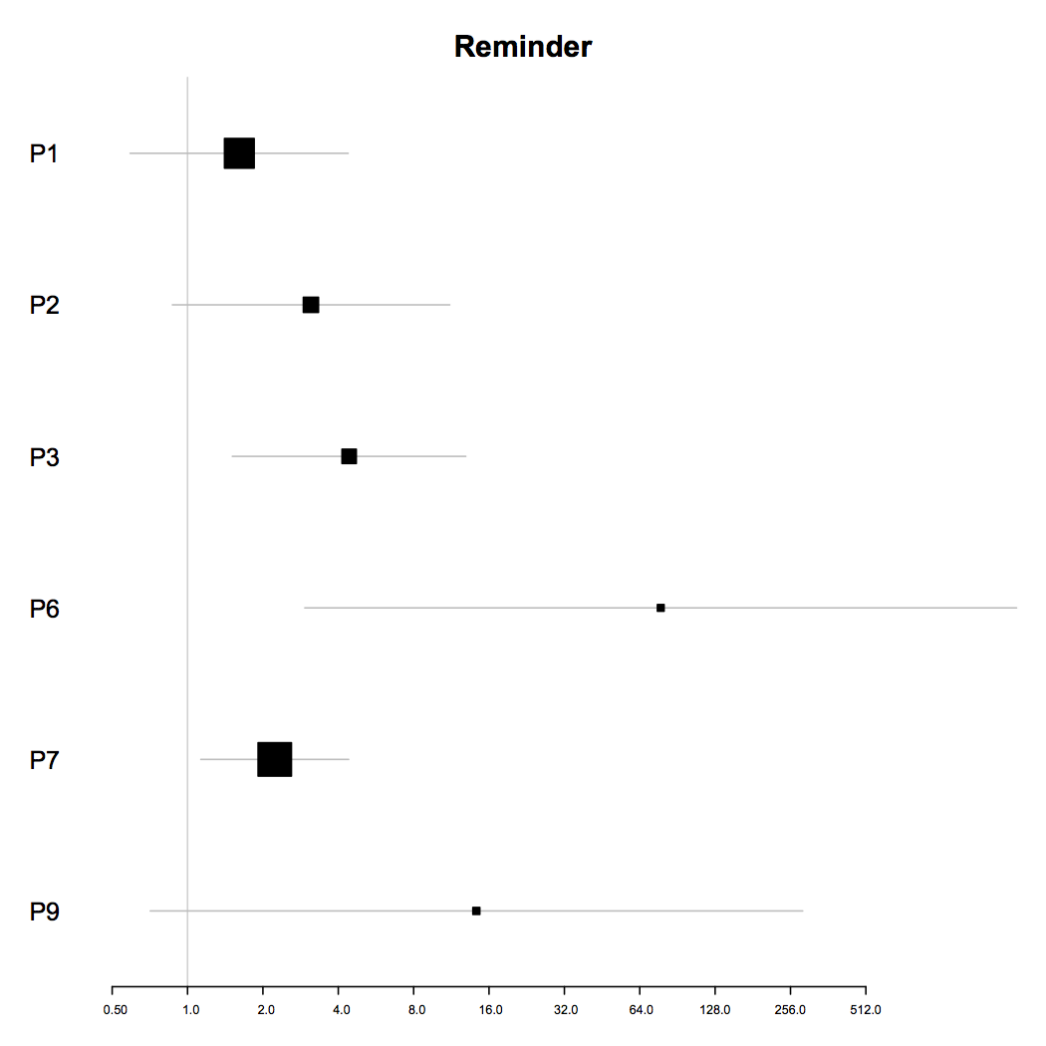
Plot of incidence rate ratios and 95% CIs (x-axis) for the association of the daily reminder and the amount of engagement for each participant (y-axis) in univariable analyses. For P4, the univariable model did not converge. P4 is hence not included in this plot. P: participant.

In univariable analyses, the daily reminder was a significant predictor of the amount of engagement for 3 participants (P3, P6, and P7; see Figure 2). In multivariable analyses, the daily reminder was a significant predictor for 1 participant (IRR=4.31; 95% CI 1.73-10.73; *P*<.01). For this participant (P3), the receipt of a reminder was associated with a 331% increase in the amount of engagement in the next 12 hours (see Table 6).

#### Motivation to Reduce Alcohol

**Figure 3:**
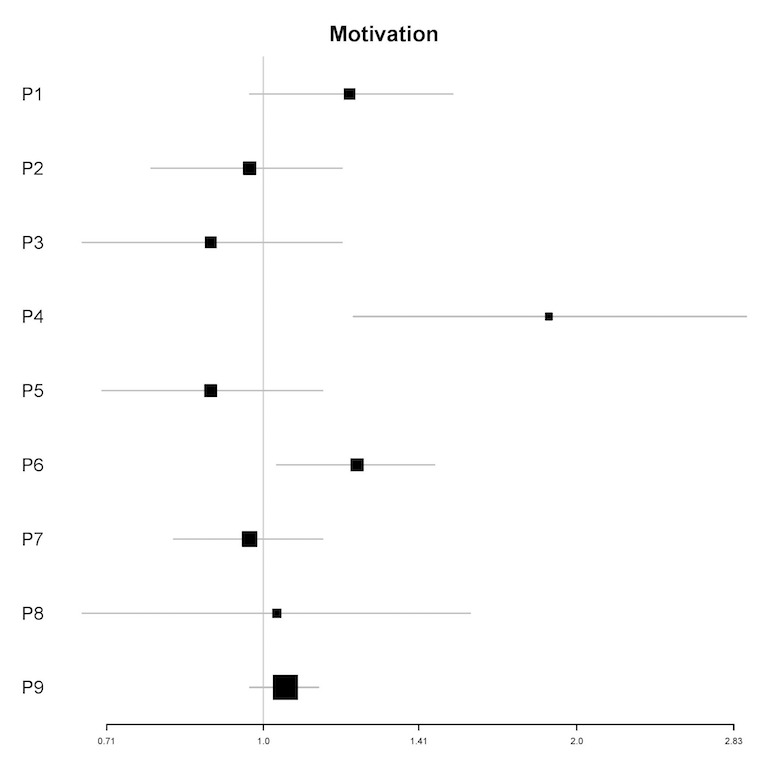
Plot of incidence rate ratios and 95% CIs (x-axis) for the association of motivation to reduce alcohol and the frequency of engagement for each participant (y-axis) in univariable analyses. P: participant.

In univariable analyses, motivation to reduce alcohol was a significant predictor of the frequency of engagement for 2 participants (P4 and P6; see Figure 3). In multivariable analyses, motivation to reduce alcohol was a significant predictor for 1 participant (IRR=1.14; 95% CI 1.02-1.27; *P*=.02). For this participant (P4), a 1-point increase in motivation to reduce alcohol was associated with a 14% increase in the number of log-ins in the next 12 hours (see Table 6).

**Figure 4:**
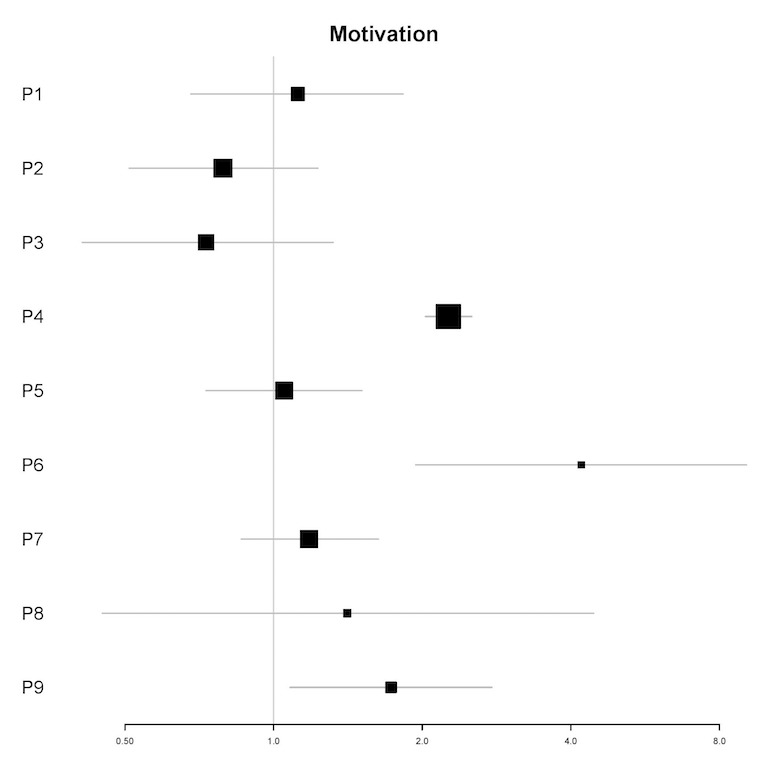
Plot of incidence rate ratios and 95% CIs (x-axis) for the association of motivation to reduce alcohol and the amount of engagement for each participant (y-axis) in univariable analyses. P: participant.

In univariable analyses, motivation to reduce alcohol was a significant predictor of the amount of engagement for 3 participants (P4, P6, and P9; see Figure 4). In multivariable analyses, motivation to reduce alcohol was a significant predictor for 3 participants (IRR=1.67-3.45; all *P*<.05). For these participants (P4, P6, and P7), a 1-point increase in motivation was associated with a 67% to 245% increase in the amount of engagement in the next 12 hours (see Table 6).

#### Perceived Usefulness of the App

**Figure 5:**
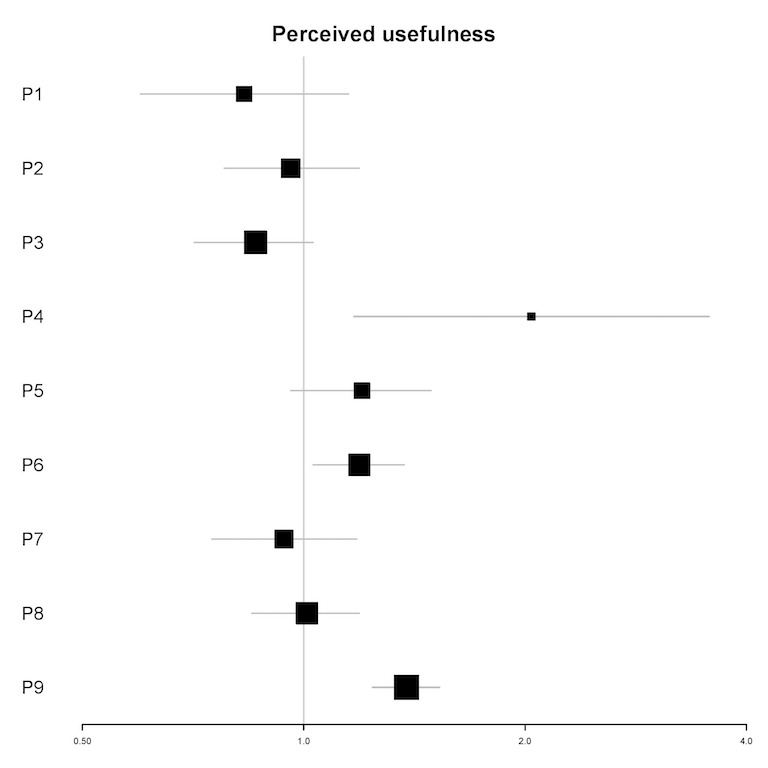
Plot of incidence rate ratios and 95% CIs (x-axis) for the association of perceived usefulness of the app and the frequency of engagement for each participant (y-axis) in univariable analyses. P: participant.

In univariable analyses, the perceived usefulness of the app was a significant predictor of the frequency of engagement for 3 participants (P4, P6, and P9; see Figure 5). In multivariable analyses, perceived usefulness of the app was a significant predictor for 3 participants (IRR=0.82-1.42; all *P*<.05). For 1 participant (P1), a 1-point increase in the perceived usefulness of the app was associated with an 18% reduction in the number of log-ins in the next 12 hours, whereas for 2 participants (P5 and P9), a 1-point increase in the perceived usefulness of the app was associated with a 38% to 42% increase in the number of log-ins in the next 12 hours (see Table 6).

**Figure 6:**
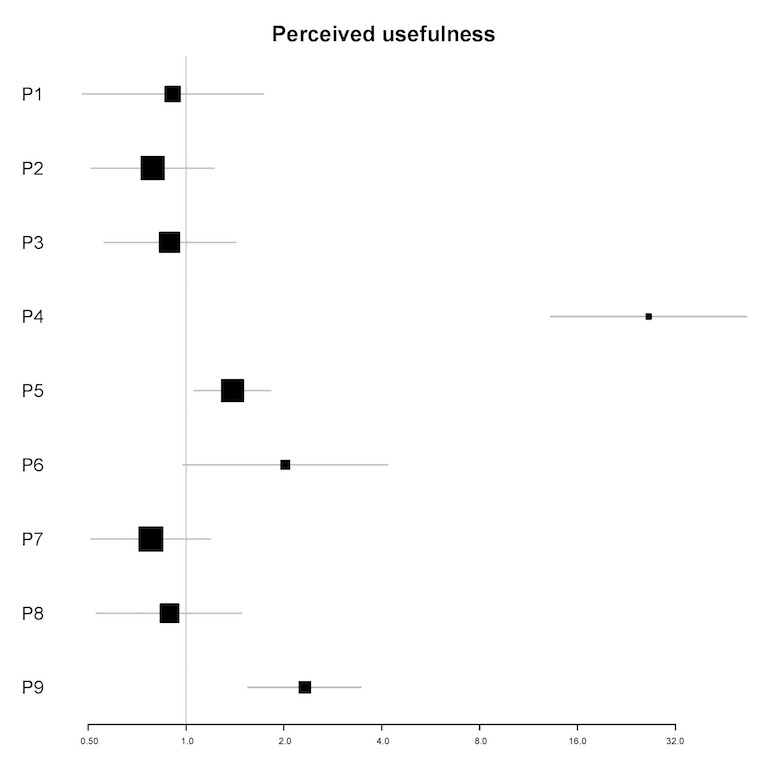
Plot of incidence rate ratios and 95% CIs (x-axis) for the association of perceived usefulness of the app and the amount of engagement for each participant (y-axis) in univariable analyses. P: participant.

In univariable analyses, the perceived usefulness of the app was a significant predictor of the amount of engagement for 3 participants (P4, P5, and P9; see Figure 6). In multivariable analyses, the perceived usefulness of the app was a significant predictor for 4 participants (IRR=0.52-137.32; all *P*<.05). For 1 participant (P7), a 1-point increase in the perceived usefulness of the app was associated with a 48% reduction in the amount of engagement in the next 12 hours. For 3 participants (P4, P5, and P9), a 1-point increase in perceived usefulness of the app was associated with a 67% to 13,632% increase in the amount of engagement in the next 12 hours (see Table 6).

#### Alcohol Consumption

**Figure 7:**
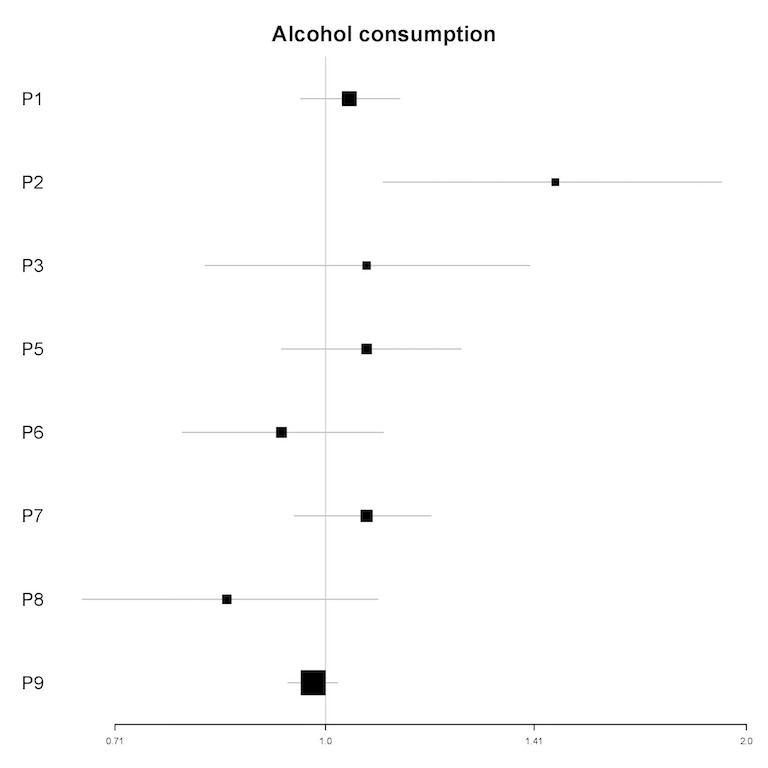
Plot of incidence rate ratios and 95% CIs (x-axis) for the association of alcohol consumption and the frequency of engagement for each participant (y-axis) in univariable analyses. For P4, the univariable model did not converge. P4 is hence not included in this plot. P: participant.

In univariable analyses, the number of drinks containing alcohol consumed in the past 12 hours was a significant predictor of the frequency of engagement for 1 participant (P2; see Figure 7). In multivariable analyses, the number of drinks containing alcohol consumed in the past 12 hours was a significant predictor for 1 participant (IRR=1.50; 95% CI 1.16-1.93; *P*<.01). For this participant (P2), each alcoholic drink consumed in the past 12 hours was associated with a 50% increase in the number of log-ins in the next 12 hours (see Table 6).

**Figure 8:**
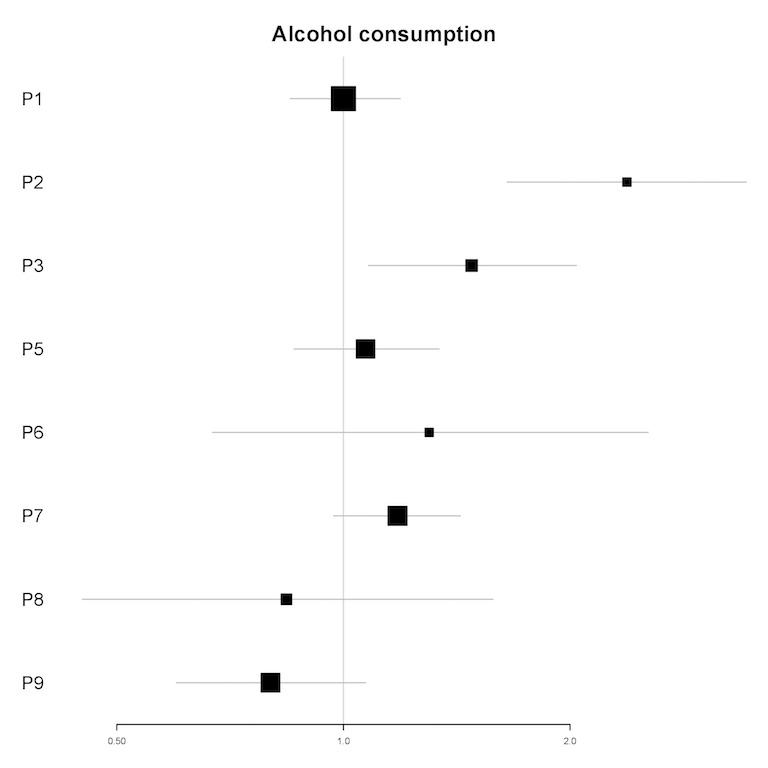
Plot of incidence rate ratios and 95% CIs (x-axis) for the association of alcohol consumption and the amount of engagement for each participant (y-axis) in univariable analyses. For P4, the univariable model did not converge. P4 is hence not included in this plot. P: participant.

In univariable analyses, the number of drinks containing alcohol consumed in the past 12 hours was a significant predictor of the amount of engagement for 2 participants (P2 and P3; see Figure 8). In multivariable analyses, the number of drinks containing alcohol consumed in the past 12 hours was a significant predictor for 2 participants (IRR=1.38-2.38; *P*<.01). For these participants (P2 and P3), each alcoholic drink consumed in the past 12 hours was associated with a 38% to 138% increase in the amount of engagement in the next 12 hours (see Table 6).

#### Perceived Lack of Time

**Figure 9:**
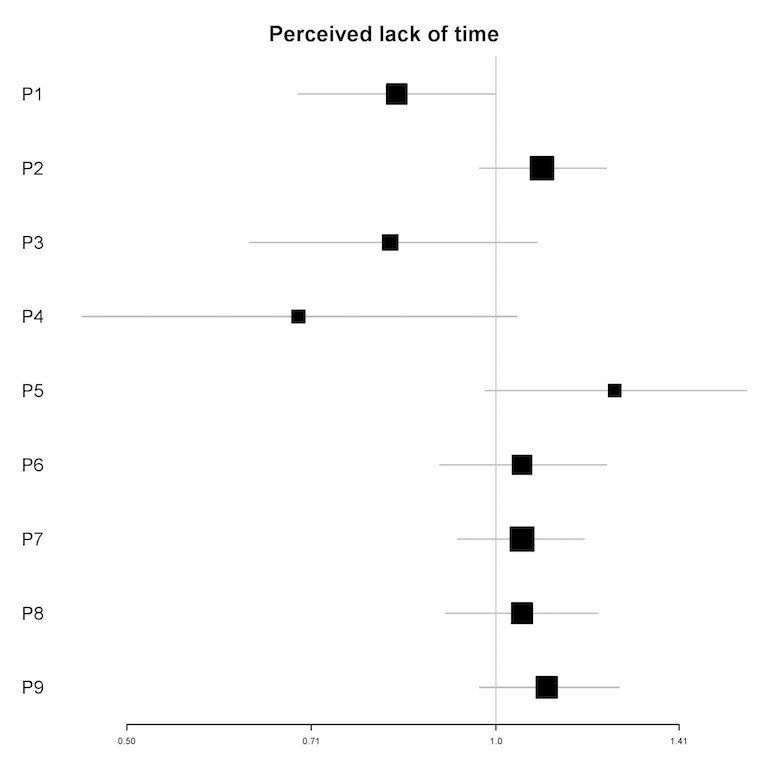
Plot of incidence rate ratios and 95% CIs (x-axis) for the association of perceived lack of time and the frequency of engagement for each participant (y-axis) in univariable analyses. P: participant.

In univariable analyses, the perceived lack of time was not a significant predictor of the frequency of engagement for any of the participants (see Figure 9). In multivariable analyses, the perceived lack of time was a significant predictor for 2 participants (IRRs=0.77-1.13; *P*<.05). For 1 participant (P6), a 1-point increase in the perceived lack of time (meaning that they had more time for the app) was associated with a 23% reduction in the number of log-ins in the next 12 hours. For the other participant (P2), a 1-point increase in the perceived lack of time was associated with a 13% increase in the number of log-ins in the next 12 hours (see Table 6).

**Figure 10:**
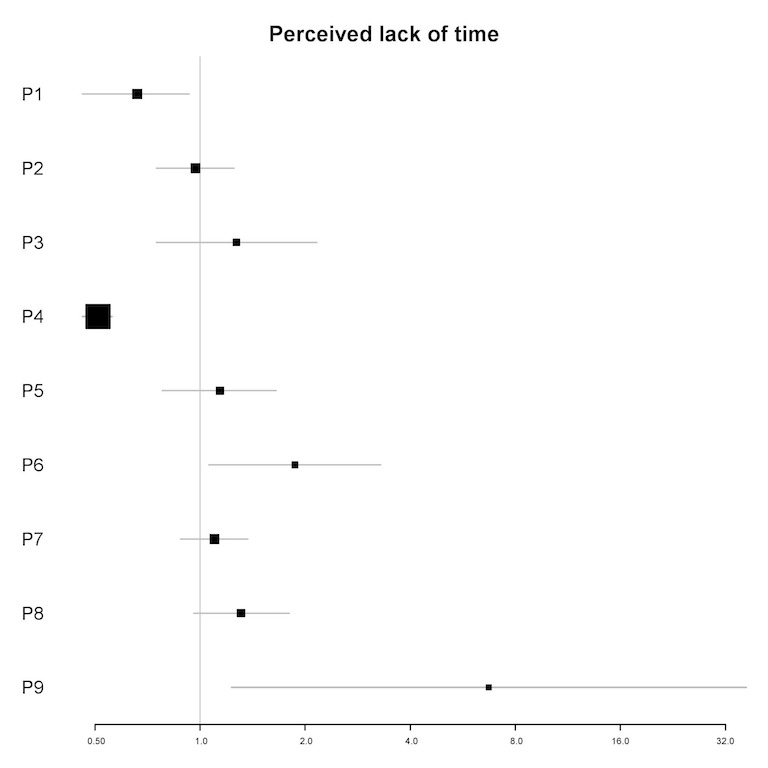
Plot of incidence rate ratios and 95% CIs (x-axis) for the association of perceived lack of time and the amount of engagement for each participant (y-axis) in univariable analyses. P: participant.

In univariable analyses, the perceived lack of time was a significant predictor of the amount of engagement for 4 participants (P1, P4, P6, and P9; see Figure 10). In multivariable analyses, the perceived lack of time was a significant predictor for 2 participants (IRRs=0.20-4.77; *P*<.05). For 1 participant (P4), a 1-point increase in the perceived lack of time (meaning that they had more time for the app) was associated with an 80% reduction in the amount of engagement in the next 12 hours. For the other participant (P9), a 1-point increase in the perceived lack of time was associated with a 377% increase in the amount of engagement in the next 12 hours (see Table 6).

### Debriefing Interviews

#### Establishing a Routine

When asked to reflect on their engagement with the *Drink Less* app, the majority of participants (P2, P3, P5, P6, P7, and P8) mentioned that they established a routine to engage with the app on a daily basis over the 28-day study. They would, for example, remember to open the app every morning upon waking or when traveling to work, or every evening when returning home after work. Some participants (who had opted to receive the daily push notification) thought this was facilitated by the daily reminder:

I’ve sort of made a habit of it now, and [I’m] probably going to continue as well.

P2

I was using it every day, because I just wanted to put the summary in for the day, even if it was a drink free day. So I would always use it.

P8

#### Purposeful Versus Purposeless Engagement

The majority of participants (P1, P3, P6, P7, P8, P9, and P10) reported that they quickly learned which features they “had to” engage with. They would only open the app for a specific purpose, which typically involved logging drinks or alcohol-free days in the calendar and reviewing their progress on the dashboard, as opposed to opening the app for entertainment:

I can just go on, quickly, input the stuff, have a check of how I’m doing against the target, and then go off it.

P7

#### Momentary Triggers and Barriers to Engagement

Most participants did not feel inclined to open the app when they were in a social setting, not necessarily because they anticipated feeling embarrassed if friends, family, or colleagues would ask about why they were using an alcohol reduction app but because they wanted to stay focused on their interactions with other people:

Not necessarily just because like: “Oh, I don’t want them to know that I’m doing it”, more just like, “I’m busy and I’m having a good time, and I’ll do it later.“

P7

Some participants (P4, P5, P7, and P9) mentioned that they thought they were more likely to open the app when feeling bored. A participant (P2) tended to open the app to combat momentary cravings to drink. Some participants (P2, P7, P9, and P10) thought they were less likely to use the app when they were hungover or experiencing low mood:

I’d sort of open the game to distract myself, and say that I should not be saying yes to everything.

P2

## Discussion

### Principal Findings

This series of *N*-of-1 studies found that the utility of app-related and psychological variables in predicting 2 facets of behavioral engagement (ie, the frequency and the amount of engagement) with an alcohol reduction app differed within and between individuals. This suggests that different strategies to promote engagement may be required for different individuals, and that such strategies may have differential effects on the various facets of engagement.

In line with findings from group-level studies [47], the receipt of a proactive reminder was significantly associated with the frequency of engagement for a few participants. However, this was not the case for all participants who had opted to have the reminder switched on. This suggests that some participants may be more responsive to prompts than others. However, for some participants, significant associations were only observed in the multivariable (and not in the univariable) analyses. As this may reflect suppression effects, results for participants with inconsistent associations across uni- and multivariable analyses should be interpreted with caution. For participants receiving the daily reminder in the middle of a 12-hour measurement period (eg, P3), it was not possible to assess whether the receipt of the reminder occurred before or after app engagement, as all predictor variables were entered into one multivariable model.

In contrast to results from group-level studies [13,15], motivation to reduce alcohol was significantly associated with the amount, but not necessarily the frequency, of engagement for some participants. For these individuals, being more highly motivated to reduce alcohol consumption may make them more willing to spend time (and perhaps also effort) on the app, provided that they have decided to open the app in the first place.

Previous group-level studies have identified a negative relationship of baseline alcohol consumption with the frequency of engagement, such that the higher the alcohol consumption, the less frequent the engagement [12,13,16]. In this study, none of the participants engaged with the app at a lower rate after sessions of heavier alcohol consumption. Instead, alcohol consumption was positively related to the frequency and the amount of engagement for some participants. It is plausible that the direction of the relationship between engagement and the target behavior may vary across individuals: while some participants may be more prone to engage when they are doing well (ie, having abstained from or consumed less-than-typical amounts of alcohol), the reverse may hold for other participants.

The variable 'perceived lack of time' has typically been explored qualitatively in interviews with participants who have dropped out of RCTs of digital interventions [13]. For some participants in the present study, this variable was significantly associated with the frequency and the amount of engagement. However, the direction of the relationships varied across participants, with some participants displaying lower rates of engagement after having indicated that they had a lot of time available for the app. It should, however, be noted that for some participants (ie, P2 and P6), significant associations were only observed in the multivariable analyses. Hence, results for these participants should be interpreted with caution. P4 (who displayed significant negative associations across both uni- and multivariable analyses) may have rated herself as having a lot of time for the app at the time of the morning or evening survey, but this might have changed a few hours later, which might have interfered with app use. More frequent EMAs may, therefore, help to detect a relationship between perceived lack of time and engagement for some participants. Alternatively, participants’ availability/receptivity to engage could be automatically inferred from their calendar or phone activity [48].

In line with findings from group-level studies [41,49], the variable 'perceived usefulness of the app' was found to be one of the most consistent predictors of both the frequency and the amount of engagement with the *Drink Less* app. The direction of the associations differed across participants; although the perceived usefulness of the app tended to be positively associated with the frequency and the amount of engagement, the reverse was observed for some participants. Again, this might be indicative of the need to capture this variable at a higher resolution (ie, more frequent EMAs). Alternatively, this variable may have been subject to social desirability. It should also be noted that for some participants (ie, P1 and P7), significant associations were only observed in the multivariable analyses.

For some participants, none of the variables assessed were significantly associated with the frequency (ie, P3 and P8) or the amount of engagement (ie, P8) in either the uni- or multivariable analyses. This raises the question as to what was driving engagement for these participants. A plausible explanation in relation to frequency, as mentioned in the debriefing interviews, is that these participants established a routine to engage with the app. If this was indeed the case, habit formation could be tested as a promising strategy to promote engagement for other users [50]. The debriefing interviews were unable to shed light on key factors that might have driven participants’ amount of engagement because it was difficult for them to introspect about momentary influences on time spent on the app (particularly as the time unit of interest was seconds rather than minutes or hours). It should be noted that although daily engagement with alcohol reduction apps, such as *Drink Less*, may be brief on average, thus making it difficult for users to introspect about momentary influences on their app use, this may not generalize to apps for other behaviors or activities. For example, apps for physical activity or mindfulness meditation, which have typically been designed to be kept open while performing the target behavior, may generate larger amounts of engagement. Hence, it may be easier for users to reflect on their daily engagement with such apps [51,52].

### Strengths

To the authors’ knowledge, this was the first study to examine within-person predictors of the frequency and the amount of engagement with an alcohol reduction app. The predictors assessed in this study were selected based on evidence from group-level studies and in-depth qualitative studies with potential users of alcohol reduction apps. Compliance with the twice-daily EMAs was high (0%-16% missing data), and the automatic recording of the outcome variables in real time ensured that participant burden and missing outcome data were minimized. This study provides initial evidence that it is feasible and acceptable to gather data in this manner and a novel time series approach (ie, GAMMs) can be successfully used to model data from *N*-of-1 studies.

### Limitations

This study was conceptualized as a series of observational *N*-of-1 studies; however, participants engaged with an active digital intervention and study materials, which included behavior change techniques known to alter cognition and behavior (eg, prompts and self-monitoring) [53]. It is, therefore, possible that both predictor and outcome variables were subject to nonrandom fluctuations that were caused by participants’ engagement with the intervention and study materials. However, as engagement with digital interventions cannot be studied in isolation, without asking participants to engage with a particular intervention and related study materials, it was not possible to overcome this particular limitation.

The study sample was almost exclusively women. As men tend to exhibit more alcohol-related problems than women [54,55], it is unclear whether the same patterns of results would be observed in a more balanced or male-dominated sample. None of the participants dropped out of the study, suggesting that they were highly motivated to take part in the research. It is, therefore, possible that different patterns of results may be obtained in samples of less committed participants. The *Drink Less* app is currently available for iOS only. As market research suggests that iPhone users tend to be more affluent than Android users [56], different patterns of engagement might be observed in a sample of Android users. Participants were all aged younger than 32 years; older adults may display different patterns of engagement. It should, however, be noted that the aim of this study was not to produce results that are generalizable at the group level. In addition, 1 participant (P7) had used the app before the study period, which may have influenced their engagement. However, as participants serve as their own controls in *N*-of-1 studies, the finding that P7 engaged more frequently with the app when she had received the daily reminder is a meaningful piece of information; it could be used to inform the development of personalized engagement strategies for this unique user.

To keep participant burden to a minimum, other facets of engagement during each log-in session (eg, attention and interest) were not assessed. This study was, therefore, unable to highlight potentially interesting relationships between the predictor variables and experiential engagement ([6]; Perski et al, in press). Moreover, many participants opted to be reminded during the first measurement period (ie, during the day). As there is more time for engagement in the daytime (as compared with the nighttime), this may have confounded the observed relationship between the receipt of the daily reminder and the frequency and amount of engagement.

### Avenues for Future Research

Descriptive plots were used to summarize the associations of each predictor variable with the key outcome variables across participants; it was not possible to pool results from the multivariable models in a meta-analysis. As time series analysis is becoming increasingly popular in the context of *N*-of-1 studies, suitable meta-analytic techniques are evolving [44], and this should be considered in future research. For studies with a greater number of participants, multilevel models (including the GAMM) can be used to estimate both within- and between-person effects [57].

Future research should test the feasibility of using both time- and event-prompted EMAs, with participants being prompted to respond to a few questions about their experiential engagement immediately after having opened the app. This would require careful piloting given the additional participant burden and unpredictability of response requests: it is possible that this might create a disincentive to open the app as participants may anticipate an additional cost directly linked to doing so. As indicated in the debriefing interviews, it is plausible that participants’ physical location (eg, being in a social setting) is negatively associated with behavioral engagement for some participants. This could be explored further by means of accessing the location sensing data from participants’ smartphones.

The feasibility and utility of just-in-time adaptive interventions (JITAIs) [58] for promoting engagement with alcohol reduction apps should be explored further. The JITAI is a type of intervention that is specifically designed to address the dynamically changing needs of individuals. JITAIs use inputs from, for example, EMAs or data collected via wearables or the phone’s location sensors to inform what type of support each individual might need in different situations or contexts. They then automatically trigger support when the system infers that the individual is in need of or most receptive to that support. In the context of the results from this study, a JITAI could, for example, be delivered when an individual’s level of perceived usefulness of the app or motivation to reduce alcohol is inferred to be below a given threshold for action, with a view to promoting the frequency of engagement.

Future research should consider the use of observational or experimental *N*-of-1 study designs as a valuable part of intervention development. Results from this study are currently being used to inform the optimization of the *Drink Less* app, involving, for example, the optimization of the content and timing of the daily reminder, with a view to promoting engagement.

### Conclusions

This series of *N*-of-1 studies found that the utility of psychological and app-related variables in predicting the frequency and the amount of engagement with an alcohol reduction app differed within and between individuals. This highlights that important within-person associations may be masked in group-level designs and suggests that different strategies to promote engagement may be required for different individuals.

## Appendix

Multimedia Appendix 1 Plots of participants' frequency of engagement over the course of the study. The y-axis displays frequency counts (ie, the frequency of engagement per 12-hour measurement period); the x-axis displays the 56 measurement periods.

Multimedia Appendix 2 Results from the univariable generalized additive mixed models.

## Abbreviations

AR: autoregressive
EMA: ecological momentary assessment
GAMM: generalized additive mixed model
IRR: incidence rate ratio
JITAI: just-in-time adaptive intervention
MA: moving average
RCT: randomized controlled trial
UCL: University College London
