# Supplementary material for: Do Daily Fluctuations in Psychological and App-Related Variables Predict Engagement With an Alcohol Reduction App? A Series of N-Of-1 Studies
Source: JMIR Mhealth Uhealth. 2019 Oct 2;7(10):e14098. doi: 10.2196/14098 (PMC6777278; doi:10.2196/14098)
Supplement: Multimedia Appendix 1 [file mhealth_v7i9e14098_app1.pdf]

Multimedia Appendix 1

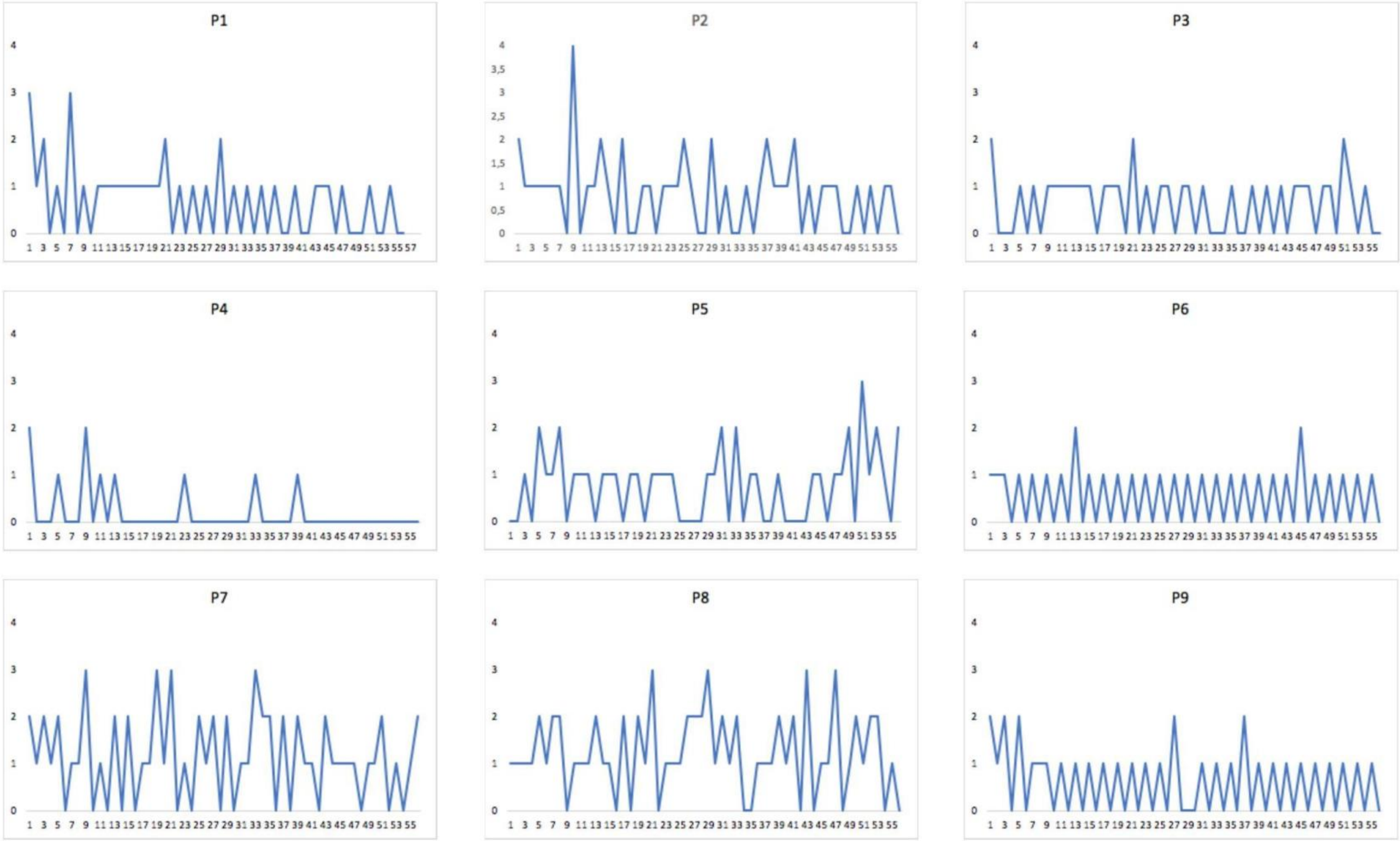

Plots of participants' frequency of engagement over the course of the study. The y-axis displays frequency counts (i.e. the frequency of engagement per 12-hour measurement period); the x-axis displays the 56 measurement periods.

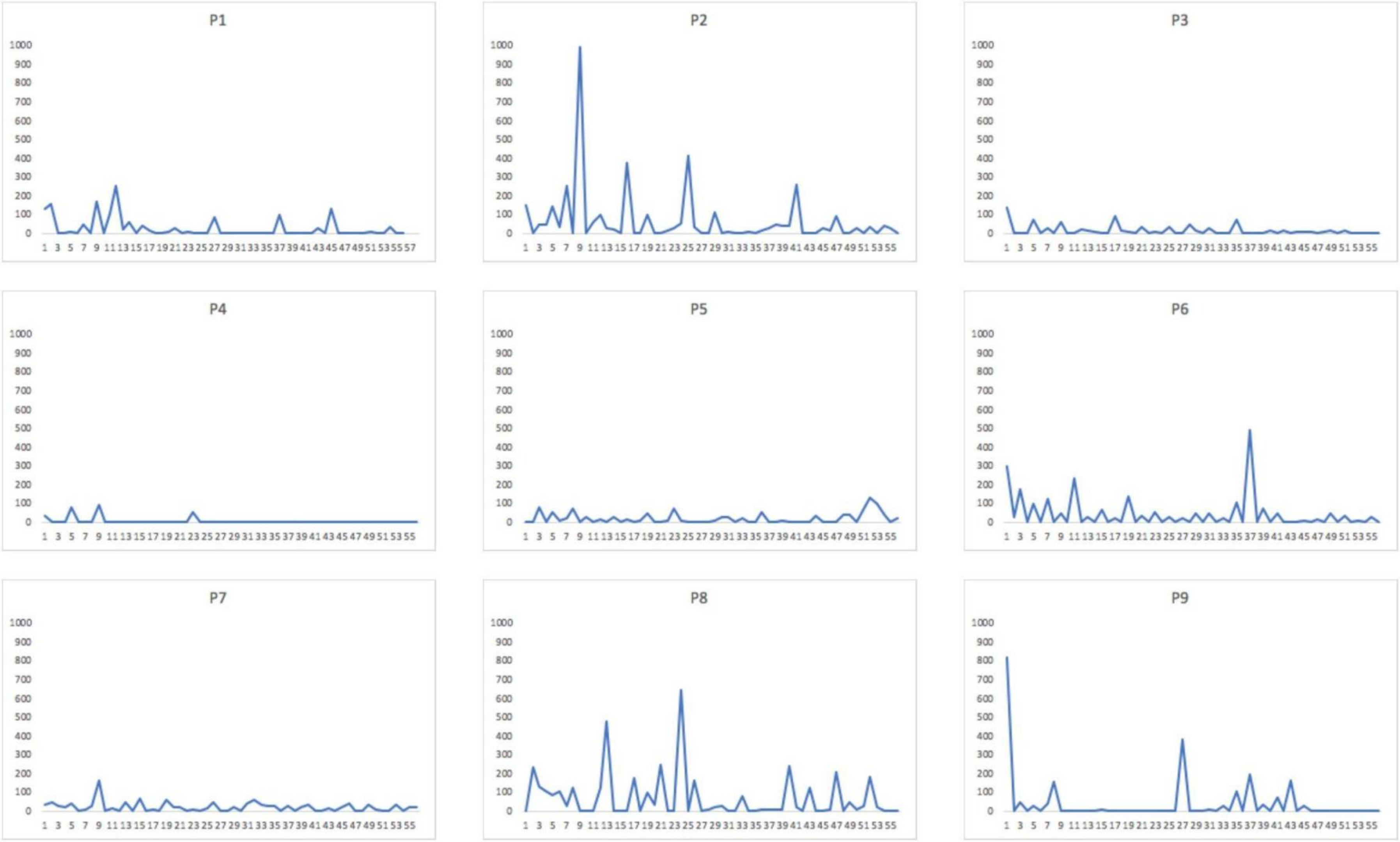

Figure. Plots of participants' amount of engagement over the course of the study. The y-axis displays time in seconds (i.e. time spent on the app per 12-hour measurement period); the x-axis displays the 56 measurement periods.

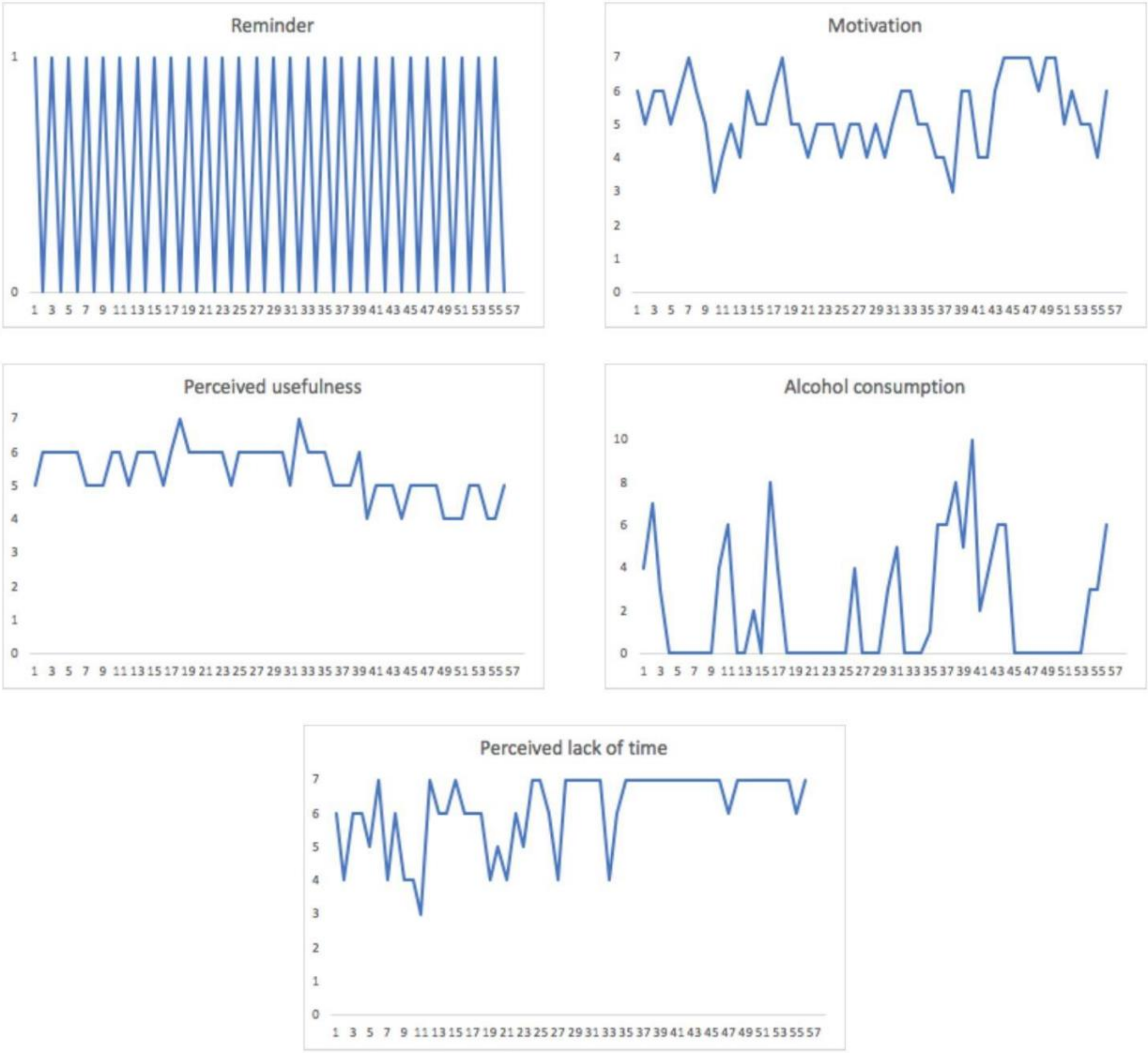

Figure. Plots of the predictor variables for P1 over the course of the study.
