# Supplementary material for: Do Daily Fluctuations in Psychological and App-Related Variables Predict Engagement With an Alcohol Reduction App? A Series of N-Of-1 Studies
Source: JMIR Mhealth Uhealth. 2019 Oct 2;7(10):e14098. doi: 10.2196/14098 (PMC6777278; doi:10.2196/14098)
Supplement: Multimedia Appendix 2 [file mhealth_v7i9e14098_app2.pdf]

Multimedia Appendix 2

Frequency of engagement

Table 1. Results from the univariable analyses of the association between the daily reminder and the frequency of engagement.

**ID**	**IRR (95% CI)[a]**	***P*-value**
P1	3.15 (1.24-8.00)	.02
P2	1.58 (0.58-4.29)	.38
P3	1.72 (0.45-6.57)	.43
P4[b]	-	-
P5[c]	-	-
P6	7.63 (0.74-79.09)	.09
P7	3.26 (2.15-4.96)	< .001
P8[c]	-	-
P9	16.76 (5.42-51.83)	< .001

[a] IRR (95% CI) = Incidence Rate Ratio and 95% Confidence Interval. [b] For P4, neither a Generalised Additive Mixed Model (GAMM), nor a Generalised Additive Model (GAM) would converge. [c] P5 and P8 opted not to receive a daily reminder.

Table 2. Results from the univariable analyses of the association between motivation to reduce alcohol and the frequency of engagement.

**ID**	**IRR (95% CI)**	***P*-value**
P1	1.21 (0.97-1.52)	.10
P2	0.97 (0.78-1.19)	.75
P3	0.89 (0.67-1.19)	.45
P4	1.88 (1.22-2.91)	.01
P5	0.89 (0.70-1.14)	.35
P6	1.23 (1.03-1.46)	.03
P7	0.97 (0.82-1.14)	.73
P8	1.03 (0.67-1.58)	.90
P9	1.05 (0.97-1.13)	.26

Table 3. Results from the univariable analyses of the association between perceived usefulness of the app and the frequency of engagement.

**ID**	**IRR (95% CI)**	***P*-value**
P1	0.83 (0.60-1.15)	.27
P2	0.96 (0.78-1.19)	.72
P3	0.86 (0.71-1.03)	.10
P4	2.04 (1.17-3.56)	.01
P5	1.20 (0.96-1.49)	.11
P6	1.19 (1.03-1.37)	.02
P7	0.94 (0.75-1.18)	.59
P8	1.01 (0.85-1.19)	.92
P9	1.38 (1.24-1.53)	< .001

Table 4. Results from the univariable analyses of the association between alcohol consumption and the frequency of engagement.

**ID**	**IRR (95% CI)**	***P*-value**
P1	1.04 (0.96-1.13)	.34
P2	1.46 (1.10-1.92)	.01
P3	1.07 (0.82-1.40)	.60
P4[a]	-	-
P5	1.07 (0.93-1.25)	.35
P6	0.93 (0.79-1.10)	.40
P7	1.07 (0.95-1.19)	.28
P8	0.85 (0.67-1.09)	.20
P9	0.98 (0.94-1.02)	.26

[a] For P4, neither a GAMM, nor a GAM would converge.

Table 5. Results from the univariable analyses of the association between perceived lack of time and the frequency of engagement.

**ID**	**IRR (95% CI)**	***P*-value**
P1	0.83 (0.69-1.00)	.06
P2	1.09 (0.97-1.23)	.15
P3	0.82 (0.63-1.08)	.16
P4	0.69 (0.46-1.04)	.08
P5	1.25 (0.98-1.60)	.08
P6	1.05 (0.90-1.23)	.52
P7	1.05 (0.93-1.18)	.47
P8	1.05 (0.91-1.21)	.52
P9	1.10 (0.97-1.26)	.15

Amount of engagement

Table 6. Results from the univariable analyses of the association between the daily reminder and the amount of engagement.

**ID**	**IRR (95% CI)**	***P*-value**
P1	1.61 (0.59-4.38)	.36
P2	3.11 (0.87-11.15)	.09
P3	4.42 (1.51-12.92)	.01
P4[a]	-	-
P5[b]	-	-
P6	77.63 (2.94-2,047.60)	.01
P7	2.23 (1.13-4.40)	.03
P8[b]	-	-
P9	14.24 (0.71-286.33)	.09

[a] For P4, neither a GAMM, nor a GAM would converge. [b] P5 and P8 opted not to receive a daily reminder.

Table 7. Results from the univariable analyses of the association between motivation to reduce alcohol and the amount of engagement.

**ID**	**IRR (95% CI)**	***P*-value**
P1	1.12 (0.68-1.83)	.65
P2	0.79 (0.51-1.23)	.30
P3	0.73 (0.41-1.32)	.30
P4	2.26 (2.03-2.52)	< .001
P5	1.05 (0.73-1.51)	.78
P6	4.20 (1.94-9.07)	.001
P7	1.18 (0.86-1.63)	.32
P8	1.41 (0.45-4.45)	.56
P9	1.73 (1.08-2.77)	.03

Table 8. Results from the univariable analyses of the association between perceived usefulness of the app and the amount of engagement.

**ID**	**IRR (95% CI)**	***P*-value**
P1	0.91 (0.48-1.73)	.78
P2	0.79 (0.51-1.22)	.30
P3	0.89 (0.56-1.42)	.62
P4	26.48 (13.25-52.91)	< .001
P5	1.39 (1.06-1.82)	.02
P6	2.02 (0.98-4.17)	.06
P7	0.78 (0.51-1.19)	.25
P8	0.89 (0.53-1.48)	.65
P9	2.32 (1.55-3.45)	< .001

Table 9. Results from the univariable analyses of the association between alcohol consumption and the amount of engagement.

**ID**	**IRR (95% CI)**	***P*-value**
P1	1.00 (0.85-1.19)	.96
P2	2.38 (1.65-3.43)	< .001
P3	1.48 (1.08-2.04)	.02
P4[a]	-	-
P5	1.07 (0.86-1.34)	.53
P6	1.30 (0.67-2.54)	.45
P7	1.18 (0.97-1.43)	.10
P8	0.84 (0.45-1.58)	.60
P9	0.80 (0.60-1.07)	.14

[a] For P4, neither a GAMM, nor a GAM would converge.

Table 10. Results from the univariable analyses of the association between perceived lack of time and the amount of engagement.

**ID**	**IRR (95% CI)**	***P*-value**
P1	0.66 (0.46-0.93)	.02
P2	0.97 (0.75-1.25)	.80
P3	1.27 (0.75-2.16)	.38
P4	0.51 (0.46-0.56)	< .001
P5	1.14 (0.78-1.65)	.50
P6	1.87 (1.06-3.29)	.04
P7	1.10 (0.88-1.37)	.41
P8	1.31 (0.96-1.80)	.10
P9	6.72 (1.23-36.75)	.03
